# Health needs and perception of health care quality among Asylum Seekers and Refugees in an Italian local health authority: A qualitative study

**DOI:** 10.3389/fpubh.2023.1125125

**Published:** 2023-04-12

**Authors:** Francesca Marchetti, Jessica Preziosi, Francesca Zambri, Gabriella Tambascia, Annachiara Di Nolfi, Paola Scardetta, Flavia Splendore, Sofia Colaceci, Maura Coia, Emanuele Caredda, Loredana Masi, Vittorio De Luca, Alberto Perra, Angela Giusti

**Affiliations:** ^1^National Center for Disease Prevention and Health Promotion, National Institute of Health, Rome, Italy; ^2^Department of Biomedicine and Prevention, Tor Vergata University, Rome, Italy; ^3^Saint Camillus International University of Health and Medical Sciences (UniCamillus), Rome, Italy; ^4^Department of Prevention, Local Health Authority “Roma 5”, Rome, Italy; ^5^General Directorate for Health Prevention, Ministry of Health, Rome, Italy; ^6^Maternal and Child Department, Local Health Authority “Roma 5”, Rome, Italy; ^7^Department of Mental Health and Addiction, Local Health Authority “Roma 5”, Rome, Italy

**Keywords:** Asylum Seeker, Refugee, health promotion, health needs, breastfeeding, infant and young child feeding, humanitarian emergencies, migration

## Abstract

**Background:**

Migrants, Asylum Seekers and Refugees (ASRs) represent a vulnerable diversified population with increased risks of developing health problems, and in the hosting countries several barriers often hamper their access to the health services. Gathering information about ASRs’ experiences and perceptions of host country health care systems may contribute to improve the quality of health care provided. The aim of this study was to explore the health needs in their bio-psycho-social meaning, and the quality of health care as perceived from the ASRs’ perspective.

**Methods:**

The qualitative descriptive study was conducted as part of the Project “G-START – testing a governance model of receiving and taking care of the Asylum Seekers and Refugees.” Through purposeful and snowball sampling, four Focus Groups conducted in English, Italian and French were carried out between July and August 2019, involving 50 ASRs hosted by four reception centers located on the territory pertaining to an Italian Local Health Authority covering a general population of 500.000 people. The analysis of data was categorical, and was performed using N-Vivo software.

**Results:**

The macro-categories emerged were the ASRs’ bio-psycho-social health needs, including mental health, sexual and reproductive health, food and nutrition, knowledge of the health care system, need for inclusion; healthcare services access, including barriers before and after the access and the ability of the local health system to respond to existing and evolving demands; strengths of the healthcare and reception systems, and suggestions for improving them in the future.

**Discussion and conclusions:**

ASRs present vulnerabilities and specific health needs, and the health care system is not always able to guarantee access or to respond to these needs. Several obstacles have been highlighted, such as linguistic barriers and lack of cultural mediation, bureaucratic and administrative barriers, lack of knowledge of the Italian health care system. An effective reorganization of services driven by a more detailed output analysis of the target population needs, together with the use of cultural mediation, peer to peer education and support, and the training of health professionals are recommended to ensure a more accessible, equitable and effective health care system at local level.

## 1. Background

In recent years there has been an increase in the migratory flows towards Europe, representing the top destination for international migrants (1). In 2020, European countries welcomed 87 million of migrants, mostly non-Europeans (i.e., over 40 million), with an increase in the phenomenon of almost 16% compared to 2015 (2). In the same period, Italy was the eleventh most popular destination in the world for migrants, and the sixth for Refugees and Asylum Seekers in Europe (2, 3). In 2021, migratory flows in Europe recorded 151,417 first arrivals, of which 114,275 by sea and 37,142 by land, and Italy, through the Central Mediterranean route, was the European country with the highest number of first arrivals, equal to 67,477, double compared to 2020 (4).

Beyond the extent of the phenomenon, its urgency also arises in relation to the social and health characteristics of this population. During the migration process migrants face stressful situations, starting from their countries of origin, as well as during the journey and arrival in the host country. This implies an increased risk of developing mental health problems (5) and higher rates of depression, anxiety and post-traumatic stress disorders than the general population (6, 7). Furthermore, the resettlement in a new country can be source of stress due to social isolation, financial problems, cultural differences and housing difficulties, factors that can negatively affect migrants’ psycho-physical health (7–10). The migration process itself, therefore, impact this population’s health, causing an increasing vulnerability. Despite that, for migrants, Refugees, and Asylum Seekers the access to the health care is often restricted in the host countries (11, 12), albeit with some variability (13), also due to linguistic, cultural, economic, and bureaucratic barriers (14, 15). This represents a great Public Health challenge that, unfortunately, has shown little progress over the years (12).

The definition of “migrant” varies among the international agencies and the terms used in literature for describing this population depend on diverse factors, such as the legal status, citizenship, and reason for migration (16). In this study, we refer to the definitions reported in Table 1.

**Table 1:** Definitions.

Term	Definition (International Agency)	Web source
Migrant	A person who moves away from his or her place of usual residence, whether within a country or across an international border, temporarily or permanently, and for a variety of reasons (IOM).	https://www.iom.int/about-migration
Refugee	Someone who is unable or unwilling to return to their country of origin owing to a well-founded fear of being persecuted for reasons of race, religion, nationality, membership of a particular social group, or political opinion (UNHCR).	https://www.unhcr.org/3b66c2aa10.html
Asylum seeker	Someone whose request for sanctuary is yet to be processed (UNHCR).	https://www.unhcr.org/en-ie/asylum-seekers.html

IOM, International Organization For Migration; UNHCR, United Nations High Commissioner For Refugees.

The welcoming procedures for migrants vary according to the host country regulations. In Italy, the reception system for all international protection seekers, including families with minors and young adults, is ensured by the Reception and Integration System (“Sistema di Accoglienza e Integrazione,” SAI), set by the Ministry of Interiors in 2020 (17). During the last decade, the government political orientation and choices had a significant impact on the structure of the reception system, the supply of resources and the provision of services to Refugee and Asylum Seekers. At present, in the Reception and Integration System most of the essential services are provided, except for health care, which is guaranteed by the Italian National Health Service.

The Italian National Health System is universalistic, and provides health care to the entire population. Since 1982, for specialist and outpatient services, some categories of medications, and non-urgent use of emergency care, a co-payment is required as a share in the health care costs. The amount of this “*Ticket*” varies according to the service: the more expensive ones will involve a more substantial expense. The “*Ticket*” is required except for specific categories of health or social conditions, for which health care is free, i.e., primary health care, maternity care, national cancer prevention programs (breast, colon-rectal and cervical cancer screening), all vaccinations included in the National Vaccination Plan, hospital care, several chronic and other health conditions. Asylum Seekers waiting for the residence permit can access to urgent and essential care through the attribution of the regional access code “Foreigner Temporarily Present” (“Straniero Temporaneamente Presente,” STP) or “Unregistered European” (“Europeo Non Iscritto,” ENI), that comprise an exemption from participation in health care costs. This regional code is valid for 6 months, and renewable. For refugees, instead, exist the obligation of registering with the National Health System and obtaining the necessary documentation for access to all services. Lazio Region established (18) the attribution of a code of exemption (“E06”) for refugees who have a regular residence permit. At the time of the Project, this exemption lasted 6 months, and could not be renewed.

The international literature highlights that information on migrants’ experiences and perceptions of the healthcare systems in the host countries could play a critical role in quality of care improving (11, 19). Understanding the experiences and problems of accessing health services of this population, in fact, has an impact on its health (20), considering that access is one of the main health indicators (21). Moreover, evidence shows that an adequate health response leads to good health outcomes not only for migrant population, but also for the host population (22).

Migrants are often under-represented in health decision-making processes (23), and European countries should critically examine their health services ability to address their health needs, also considering their perception of the health care received. This could lead to the development of new strategies to provide migrant-sensitive health services and systems (24). Furthermore, the studies exploring migrants’ own view on health needs and health care access are still exiguous, and more research on this topic is needed (13).

The main objective of this study, therefore, was to explore the bio-psycho-social health needs, and the perceived quality of health care, health promotion and prevention activities from the Asylum Seekers and Refugees’ (ASRs) perspective.

This study was carried out as part of the Project “G-START - Governance, Health, Territory, Reception for Asylum Seekers and Refugees: testing a model” (“G-START: Governance, Salute, Territorio, Accoglienza per Richiedenti Asilo e Titolari di Protezione”) (25, 26), developed by the Italian National Institute of Health in partnership with the Local Health Authority (LHA) “*Azienda Sanitaria Locale (ASL) Roma 5*” and the International Organization for Migrations (IOM), promoted by the Ministry of the Interior and the European Union. The Project has received funding from the European Asylum, Migration and Integration Fund 2014–2020 under grant agreement PROG-2261, and aimed to strengthen the first and second reception system and the health protection of Asylum Seekers and Refugees. The results of this qualitative study were then meant to guide action during the 3 years of project duration and to provide key elements for sustainability.

## 2. Materials and methods

The study design was qualitative descriptive (27). A series of Focus Group (FG) was organized as part of the Project “G-START.” The FGs were conducted in the first and second accommodation facilities (reception centers) located on the territory of the LHA leader of the Project that covers a general population of 500.000 people.

A purposeful and snowball sampling was used to recruit the participating ASRs, contacted by the Project partners through the reception system network. To facilitate interactions and discussions, the research team applied homogeneity criteria in recruiting participants for each FG. Each participant signed a written informed consent and reported socio-demographic data on an anonymous self-administered form.

The discussion was facilitated by experienced researchers of the Italian National Institute of Health on the basis of a grid of semi-structured questions shared with the Project group (Table 2). An observer from the project team was also present during each FG. The FG discussions lasted 90–120 min and were conducted in English, Italian and French and, in some cases, the simultaneous translation in Arabic and Farsi was provided by cultural mediators. All the FGs were digitally audio-recorded and fully transcribed. The data analysis was categorical: categories were developed both deductively, starting from the research question, and inductively, based on the emerging themes. The analysis was conducted using N-Vivo software. The study protocol was approved by the LHA Ethic Committee.

**Table 2:** Semi-structured focus group (FG) questions.

Aims	Questions
Main health needs	Which are, in your opinion, the health needs of ASRs, understanding health in a broad sense, as psychological, physical, moral and social wellbeing and not only as absence of disease?
Describe the main health problems and health determinants	Which are the main health problems you have encountered? Which are the causes or determinants of these problems?
Describe, for primary or secondary reception system, the current response to health needs, the strengths and weakness and areas of improvements	How the current organization of the reception system responds to health needs of ASRs: What are the strengths? What is working? What are the weaknesses? What could be improved?
Describe, by the point of view of health service, the current response to health needs, the strengths and areas of improvements	In which way the current health services respond to the health needs of ASRs: In the management of the situations that have occurred until now, what were the strengths? What worked? In the management of the situations that have occurred until now, what were the weaknesses? What could be improved?
Other suggestions	Do you have any suggestions to improve health care of ASRs?

## 3. Results

From July to August 2019, four FGs took place. The participants where men, women, women victims of trafficking and families hosted in primary and secondary reception centers located in the territory of the LHA “*ASL Roma 5*.” In particular, two FGs were organized with men, one with women, one with women victims of trafficking, and one included families with children.

Fifty ASRs participated in the FGs. Of these, 25 were men and 25 women, with an average age of 29.8 years. The main socio-demographics characteristics are presented in Table 3.

**Table 3:** Socio-demographic characteristics of Asylum Seekers and Refugees (ASRs).

Participants	*N* (%)
Women	25 (50)
Men	25 (50)
Nationality	
Nigeria	24 (48)
Egypt	4 (8)
Pakistan	4 (8)
Afghanistan	3 (6)
Gambia	3 (6)
China	2 (4)
Iran	2 (4)
Algeria	1 (2)
Guinea	1 (2)
Russia	1 (2)
Somalia	1 (2)
Tunisia	1 (2)
Turkey	1 (2)
Missing	2 (4)
Mother language (more than an option)	
English	28 (56)
Arabic	5 (10)
Farsi	4 (8)
Urdu	4 (8)
Chinese	2 (4)
Ika	2 (4)
Mandinka	2 (4)
Kurdish	1 (2)
Dari	1 (2)
French	1 (2)
Jola	1 (2)
Pulaar	1 (2)
Russian	1 (2)
Somali	1 (2)
Turkish	1 (2)
Bridge-language (more than an option)	
English	33 (66)
Italian	29 (58)
Arab	3 (6)
Chinese	2 (4)
French	2 (4)
Pulaar	1 (2)
Urdu	1 (2)
First reception in Italy/Europe	
2013	1 (2)
2014	2 (4)
2015	3 (6)
2016	15 (30)
2017	11 (22)
2018	7 (14)
2019	6 (12)
Status	
Asylum Seeker	25 (50)
Refugee	24 (48)
Repeated asylum applicant	1 (2)
Family condition	
Married/with partner	18 (36)
With children	14 (28)
Number: 1–5	
Age of children: 2 weeks–21 years	

The FGs allowed the collection of ASRs’ opinion on their health needs, quality of care and prevention/health promotion activities. From the categorical analysis of data, the following macro-categories emerged.

### 3.1. Macro-category 1: ASRs’ bio-psycho-social health needs

One of the emerged categories was the identification of ASRs’ health needs, framed in a bio-psycho-social perspective.

#### 3.1.1. Need for inclusion and daily life

A crucial aspect of the participants’ wellbeing concerned the conditions of their daily life. In fact, the ASRs’ legal status and the long waiting times for obtaining the residence permit and other personal documents made impossible for them to be fully integrated into society. For this reason, their daily life was characterized by “*suspended and empty time*” and the perception that their life was wasted.

*“For some people I saw here… five years, they sitting here, it’s enough! Like… in five years you will get three children! Like, you have a life!”*

*“All day: eating and sleeping, nothing else!”*

This stalemate condition was associated with the impossibility to realize projects and dreams and the inability to move and travel freely.

The time needed to obtain the necessary documents to start a “normal” life were very long and exhausting, and the appointments with the relevant offices were often subject to extensions.

Furthermore, not having a job and not being able to find it due to bureaucratic barriers, lack of personal documents, and employers’ prejudice, resulted to be a great challenge, and contributed to the problem of having too much free time.

Life in the reception centers was not always simple, also because *“[The center] is not like a home.”* In particular, the participants suffered the lack of privacy and personal spaces for their belongings:

*“There is no key, because even everybody wardrobe uses the same key. The rooms are not locked. The available things you take it up with yourself or you don’t see it again.”*

During the reception in Italy and in their everyday life, some of the FGs participants reported the perception of racism, discrimination and prejudice by the Italian citizens, especially towards *“blacks,”* those who came from African countries.

#### 3.1.2. Psychological and mental health

All of these conditions related to the ASRs’ legal and social status had a notable impact on the participants’ health, in particular on psychological and psychosocial wellbeing.

*“The long time I spent here has really had great effect on me, psychologically. All this waiting time is affecting [our health].”*

During the FGs emerged several references to depression (*“I’m been getting negative, negative, negative, constantly”*), stress (*“I am so tired and stress”*) anxiety and overthinking.

The psychological distress and the overthinking frequently led to insomnia problems, impacting both the quality of sleep and the daily activity of those affected.

For one man, inability to sleep had such an impact that it led him to engage in health-threatening behaviors, such as alcohol consumption:

*“Since I can’t sleep at night, even when I try as much as possible, I’ve even caught the habit of going to some market to buy wine and [alcoholic] drinks, so I could feel a little tipsy, so I could sleep. If I have to take alcohol for me to sleep, I think it’s a problem.”*

#### 3.1.3. Life styles

Despite what has just been reported, in general, alcohol addiction has not been highlighted as a widespread problem, also because *“you cannot drink in the reception center,”* but rather as a use as a relief valve in the face of daily difficulties. Moreover, drug use has only been reported in isolated cases.

Physical activity was performed by a very low percentage of participants, also due to the absence of adequate spaces such as gyms, and soccer fields. In a reception center, the participants used to play football in the near soccer field, until the Municipality avoided its use. Among the good practices, some women reported they used to organize walking groups.

#### 3.1.4. Food and nutrition

The quality of food and nutrition was a cross-cutting category among all the FGs. The participants strongly highlighted some critical issues concerning the meals in the reception centers. First of all, the lack of variability and cultural sensibility of the diet: in many cases the participants would have liked to be involved in the preparation of meals so that they could also use products and foods of their culture of origin.

*“Pasta! Pasta every day! And sometime rice with water [risotto]”.*

*“In Africa we don’t eat the same food over and over again in the morning, afternoon, evening… no, that’s not possible! We need a balanced diet.”*

The food quality and taste was often perceived to be poor. In particular, one of the problems concerned the catering logistic, according to which the meals were distributed at times distant from the consumption and, in some cases, in single plastic packages, which were then reheated on site with a microwave. For example, one participant reported that food for the whole day (lunch and dinner) was delivered to the reception center by the catering service at 7:00 in the morning.

The participants reported some perceived difficulties in accessing adequate nutrition for specific health problems (such as diabetes, hypertension, other diseases) and considered that the diet was affecting their own health. During the discussions, in fact, emerged the feeling of being unwell and unhealthy: because of the food, the *“belly is heavy”* and the body is *“without blood,”* participants felt tired and, after sleeping, they felt bad. This perception was contrasted with the condition of well being given by the diet of the country of origin, which was *“full of vitamins”*:

*“When you eat good food you are healthy, but when you don’t eat good food there is no medicine that can solve the problem.”*

#### 3.1.5. Infant and child feeding

The lack of cultural sensitivity also emerged with regard to infant and young child feeding practices, in particular as regards complementary feeding - the beginning of the consumption of solid foods. According to the participants, the pediatricians taking care of the children in the centers prescribed a complementary diet that included the use of Italian foods rather than the usual ones of the family/mother’s culture of origin (e.g., African food). On the other hand, the participants very often breastfed according to the international health recommendations (6 months exclusive breastfeeding and continued breastfeeding for 2 year and beyond):

*“I have three children and have breastfed all three.”*

*“Because mother’s milk is very good for the baby!”*

#### 3.1.6. Sexual and reproductive health

Some inappropriate care practices about antenatal care emerged. As an example, some women described the prescription of several unnecessary tests and ultrasounds by the private gynecologist during physiological pregnancies. The women perceived this overtreatment as normal for pregnancy care, and were therefore surprised at how much they had to pay for care. The high costs for obstetric care were associated with the inappropriate prescriptions and, in some cases, with the need to turn to private facilities due to too long waiting lists at public services.

*“Yes, the doctor prescribed it [the test] for me. He said: - You have to pay for this, you have to pay for that…-”*

One woman reported her bad experience of hospital birth, telling that she did not feel welcomed and listened to by the healthcare personnel:

*“From the morning I had pain, pain in my belly, and I was crying, crying. They [the doctors of the hospital] came and said to me: “Ok. Caesarean section!” And me: “No, no!” and I was crying. “No cesarean? If you don’t accept, you or your child will die. Choose one”. I was scared, so scared.”*

With regard to women’s health care, participants were taken care of by local health services. However, there was a lack of knowledge about the services provided free of charge by the health facilities, in particular concerning the cervical screening. It was challenging for women to access the different kinds of contraceptive methods: on one hand, due to the unavailability among the local health services (as for the intra-uterine devices, IUD); on the other hand, because they were not accessible for free (like estrogen-progestin pills).

*“When you go there [to the local health service], they don’t give you the [estrogen-progestin] pills… they give you the name of the pills, and you have to buy it yourself.”*

Concerning Feminine Genital Mutilations, only one woman raised the issue, asking for information.

#### 3.1.7. Lack of knowledge of the Italian health care system

During the FGs a dearth of knowledge on the organization and functioning of the Italian National Health Care System and on the different levels of care provided emerged. In particular, some participants were not fully aware of the available universalistic and free health care services (e.g., the general practitioner) or asked if they needed a health insurance. Furthermore, there was some confusion about whether or not they had to pay for health care: the differences between private paid services, public health services requiring a contribution (“*Ticket*”) to the overall cost, and universalistic health care were not always clear.

Furthermore, regarding the use of emergency health services, one participant stated that, when she had been ill, the operators of the reception center had not allowed her to call an ambulance, and this attitude was been perceived by her as a lack of attention:

*“If somebody is sick, they [the operators of the reception center] are supposed to call an ambulance!”*

The waiting time for getting an appointment or an exam was often perceived by the ASRs as a consequence of prejudice and discrimination, instead of the standard functioning of the waiting list system.

In another case, the perception of *“feeling discriminated”* was probably associated with a lack of understanding of the health condition. A man was isolated due to Tuberculosis (TB), and he said that the doctor did not come to his room and *“stay with”* him.

Lack of knowledge of health practices, not supported by adequate cultural mediation, has often led to a lack of trust in the system and a poor adherence to the treatments and health practices prescribed. This was closely associated with health beliefs, cultural aspects and transcultural issues. For example, the acceptance of blood sampling was challenging for some participants. One woman, talking about pregnancy testing, said:

*“When I have to take a blood sample, they do like this: tack! And take the blood, like this: (exaggerated sucking noise) … four tubes, five tubes! Too much blood! If you are pregnant, you can’t lose all that blood!”*

### 3.2. Macro-category 2: Healthcare services access

The second macro-category that emerged from the analysis was the barriers to the access to health care services for ASRs and the ability of the local health system to respond to existing and evolving demands.

#### 3.2.1. Barriers to accessing healthcare

In more than one occasion, the participants complained about the distance to public health facilities, including the hospital, and the difficulty of reaching them by public transport, that was often not efficient. This barrier particularly affected families and parents when their children needed specialist health care: they had to travel to get to the hospital located in the center of the main city adjacent to the Municipality where they lived, and it took a lot of time and efforts. The problem of the isolation of the Municipalities in which the reception centers were located has also emerged with regard to children’s access to the school.

The access to the health services was difficult due to the waiting lists: getting an appointment or booking an examination often requested months of waiting. Moreover, the participants reported that they experienced frequent changes in the appointment date or timing, and poor punctuality of the visits. In some cases, the ASRs’ perceived this barrier as discriminatory and limiting their health access.

*“I am not happy because I went to the hospital and they told me to come back tomorrow, and the day after tomorrow, and tomorrow, and the day after tomorrow…”*

The bureaucratic procedures had a great impact on the healthcare access for the ASRs interviewed. This barrier concerned, for example, the assignment or change of their General Practitioner. The participants reported from the complex bureaucratic tangle caused by the association between the standard procedures and the problems in getting or renewing their personal documents, such as residence permits, identity cards, exemption certifications. This leaded to difficulties in accessing services, including the possibility of independently purchasing medications at the pharmacy.

*“If you go somewhere and they tell you “Go there”, and then you go there, but you cannot be received because you need papers, and you still not got them, you have to go back, and this is confusing!”*

As another aspect of this topic, the participants had the perception that bureaucracy is pervasive in all the procedures of the system. They did not understand why in all the interactions with services and professionals, they previously had to sign a form or a register, and they did not accept it willingly:

*“If I want to talk with the social worker, if I want to talk with the doctor, they never listen to you, they say: - Sign here! -”*

According to the Italian reception system, the ASRs received at their arrival in Italy an exemption from contributing to the total cost of the health services. One of the main problems in accessing health care resulted to be the expiration of this exemption, foreseen after 6 month from the activation. In fact, their only source of income consisted of the “pocket money” received by the reception center, which was not enough for the requested expenses. Moreover, due to the lack of knowledge of the Italian National Health System, in many cases the difference between paying the total cost of the health service, and paying only a part of it (the “*Ticket*”) was not clear to the participants. In this regard, dental care was often too expensive to be reachable by the ASRs.

*“I think this is the major issue, about the exemption. Because after six months it expires, and you have to pay every visit.”*

The approach to health services and healthcare professionals presented several challenges for the ASRs.

First of all, the language barrier and the lack of cultural mediation: the communication inability made interactions between the healthcare provider and the person/patient very difficult, not only because the latter did not speak Italian, but also due to the health personnel’s lack of knowledge of bridging languages such as English.

The perception of being taken care of by the healthcare system and professionals was generally lacking. In addition, the lack of continuity of care between different providers and services and the lack of tailored information led to fragmented and not clearly oriented healthcare pathways, from the participants’ perception. In many occasions, the participants reported that they did not feel welcomed and listened to by healthcare professionals:

*“I have a family doctor, but he never listens to you.”*

*“When you try to express your feelings, no one is listening. I don’t like that.”*

In other cases, they felt treated differently from Italian citizens and victims of racial discrimination.

Sometimes the ASRs who participated to the FGs did not recognized good support in health issues from the reception center and its operators. One man said:

*“I was sick, but I went to the doctor’s appointment alone. But as I was leaving I passed out, someone on the street called an ambulance and I was hospitalized. And I called them [the reception center operators] to warn them that I was in the hospital. I’ve been there for, like, two days. But when I was discharged, no one came even to pick me up at the hospital. So, they don't care about the health of the people who stay here [the reception center].”*

### 3.3. Macro-category 3: Strengths and suggestions for the future

The third macro-category included the strengths of the healthcare and reception systems, and suggestions for improving them in the future.

The strengths of the reception and health system were reported, i.e., the psychological support provided by specialists:

*“We had the psychological help, which worked very well!”*

A sense of gratitude towards the reception center and the personnel, and for receiving their care emerged from some participants.

Furthermore, in some cases the difficulties in accessing healthcare services and the lack of continuity of care were overcome by individual professionals who had specific sensitivity and skills, essential for the ASRs.

As for the suggestions for the future, the participants proposed: the institution of educational courses on proper nutrition, lifestyles, and contraception; the provision of free contraceptive within the reception centers; the extension of the exemption for the health care services; to find a solution to the bureaucratic delays and to allow the people welcomed not to waste their lives just “waiting.”

## 4. Discussion

One of the main objectives of the “G-START” Project was to strengthen the health protection of the ASRs, structuring a systemic, sustainable and long-lasting response to the psychosocial and health needs of the ASRs, beyond the end of the Project. This included a set of actions, e.g., to set up a trans-sectoral Board for health and reception (“Tavolo Salute e Accoglienza,” TASAC), to carry out a Situational Analysis with all the Municipality and reception centers, and to provide an integrated governance of health, social and community services. The first step was a quali-quantitative assessment, which has been realized both through FGs, and in-site visits/quantitative data collection. From the analysis of the ASRs’ perceived health needs and health care, several macro-categories and categories emerged.

Considering the health in a bio-psycho-social framework, the health needs reported by the participants also concerned aspects and conditions of their daily life. Among these, the inability of the ASRs to be included in society due to their legal status has been one of the most important issues affecting mental health. The urgency to thrive emerged from this study could be also explained by the average age of the participants and their strong motivations for life change that led them to decide to move from their country, often leaving their relatives and facing long and perilous journeys. Therefore, the perception of being stopped in this path may have been hardly acceptable to them. Previous studies described this situation of uncertainty as a “limbo” (28, 29) characterized by empty time and long waiting, and influencing men and women wellbeing and health. The asylum application and the following renewal of the residence permit are directly associated with stress and mental health consequences (29, 30). According to this, some author declared that migrants who have a job and a stable accommodation have better health outcomes, while instability and uncertainty may lead to develop severe mental disorders (30, 31). Therefore, legal status is one of the most significant factors determining access to an adequate health care for migrants in a country (16).

The most common psychological and mental health problems reported by the participants were related to depressive and anxiety symptomatology, often addressed as stress, and expressed as overthinking. Depressive disorders are widely described in the literature for the ASR population, independently or associated to Post-Traumatic stress Disorder (PTSD) (13, 30, 32–36); overthinking has been considered part of PTSD symptomatology, as well as an “idiom of distress” (37), culturally shaped in many social and cultural contexts (38). Moreover, poor sleep quality and sleep-disturbances seem to be a common health problem, and one of the possible signs of mental distress (39), which has been included among sentinel symptoms in the Italian National Guidelines for the psychological assessment, treatment and rehabilitation of international torture and violence victims (40), adopted also at a regional level in Lazio Region Guidelines (41).

As for life styles and sport, in this study the lack of ASRs’ physical activity was mainly due to structural barriers including lack of funds and resources, also reported by previous studies (42). Moreover, sedentary lifestyle has caused and was caused by having nothing to do and mental health issues, becoming an unhealthy vicious circle.

Food and nutrition were another important macro-category. The needs of the participants were related to the opportunity to access both a various and healthy diet, and foods belonging to their culture of origin. As regard the first aspect, some ASRs showed a good awareness of the role of diet for health, emphasizing the importance of consuming all the categories of the food pyramid in daily meals (especially for participants with diabetes and other pathologies). In addition, they complained about lack of taste and quality of the food, as in other studies described in literature (29, 43). With regard to the second aspect, it is important to remember that food is not only relevant for nutrition and health, but is also a component of the cultural and emotional wellbeing (44). During their displacement experiences, migrants often have to adapt to new life habits of the host or transit country, including new foods habits and environment (45), and this process of adaptation and transculturation (46) could be challenging. Food represents an element of continuity with the past, providing a bridge between the familiar and the unfamiliar (47). This could be particularly significant for mothers and families caring for their infants and children feeding.

The results of this study highlighted that cultural sensitivity in the community pediatricians’ indications on complementary feeding, which included only the use of Italian foods, needs to be improved. In contrast, as also described in the study by Joseph et al. (48), mothers would choose the staple foods of their countries of origin as the first solid foods to give to their children. This aspect is in line with the guidelines of the World Health Organization, which confirm the value of “family foods” in complementary feeding (49). The ASRs women participating to the FGs were aware of breastfeeding health benefits, and considered it as the norm, in accordance with other previous studies (48, 50, 51). In several cases they breastfed accordingly to the international recommendations (exclusive breastfeeding for the first six months, followed by the introduction of appropriate complementary feeding while continuing breastfeeding for two years and then, accordingly to the mother and/or infant desire), especially if coming from African countries (50). As reported in the literature, migratory process can influence breastfeeding and infant feeding practices, also depending by the country of origin and of arrival (50). Anyhow, migrant women are still more likely to breastfeed compared with native mothers of the host countries (52–54). This confirm that the “healthy migrant effect” (55–57) applies also to breastfeeding practices in Italy and that, in the absence of substantial policies for the protection, promotion and support of breastfeeding, the “exhausted migrant” effect is to be expected in the coming years.

Migrant women represent a highly vulnerable group in term of sexual and reproductive health (58). This study investigated this topic only partially, not addressing in depth issues such as pregnancy and delivery outcomes, and perinatal health. Nevertheless, it highlighted a lack of knowledge of the available free-of-charge antenatal and mother–child care services, which impacted the ability of the ASRs to reach the system, as described by Fair et al. (59). The cost of care (59) was another factor influencing access to health care when it was inappropriately prescribed (and therefore not foreseen by the universal National Health Care System) or when it was provided by private facilities/professionals, in some cases due to long waiting times in the public sector.

Among the sexual and reproductive health needs of the ASRs, this study confirms the poor access to contraception (60) and the lack of information and use of preventive care, such as national cancer screening programs (13, 60, 61).

As expected, a lack of knowledge of the Italian health care system emerged from the analysis of the FGs. Poor health information, and the ability to understand and use it are common in the migrant population (62), who are often unaware of their entitlements (13). The over-use of emergency services described by other studies (13, 29, 32) did not strongly emerged in this qualitative study, but was reported during the context analysis carried out at the beginning of the project and by previous studies conducted in the same LHA (63). The low level of Health Literacy adds to the vulnerability resulting from the migration process and contributes to increase it (64).

Other studies reported experiences of migrants’ positive strategies of adaptation and acculturation (65, 66). Although the study had among its aims the identification of the positive resilience strategies of ASRs, these aspects emerged only partially during the FGs and the participants mainly focused the discussion on the improvement needed in the reception and healthcare system.

Although the migrant population presents specific vulnerabilities, predominantly linked to the migratory process (67), and have a great need for health care, their access to the health system is commonly hindered or reduced: even when health care access is guaranteed under legislation, many barriers have been identified (16). This study identified several barriers limiting the health care access already described by other authors in the literature, outlining a system of care that is “hard-to-reach” (68). First of all, the barriers prior to access and the characteristics of the services that make them difficult to be reachable were described by this study. These include: the distance between services and reception centers, often located in isolated areas (16, 29); bureaucratic and administrative barriers, including the waiting times for obtaining personal documents (13, 16, 32, 69, 70); the health care costs (13, 16, 29) due to inappropriate prescriptions, the need to turn to private facilities due to too long waiting lists at public services, and the expiration of the ASRs’ exemption from participation in health care costs. The challenges related to the temporariness of the free access to healthcare, that at the time of the study lasted 6 months and could not be renewed, were widely highlighted by the participants of this study. In the years following the Project something changed, and the Lazio Region appropriately extended the duration of the exemption for refugees until obtaining a residence permit (71).

The barriers in approaching health services identified by our study, were also described in previous literature: linguistic barriers (13, 29, 69, 72) and lack of cultural mediation (73, 74), leading to lack of understanding and adherence to proposed healthcare practices or treatments; ASRs’ perception of discrimination and of “not being treated as human beings” (28); lack of continuity of care (72). All these issues can be included in a generic lack of cultural competence of the public health system.

The ASRs’ perception of the barriers described above have probably been amplified by the poor knowledge of the health system of the host country: while the waiting time to specialist health care, for example, affects migrants and Italian citizens in the same way, some practices as choosing the General Practitioner or accessing community health services are influenced by the user’s ability to navigate within the system itself, reach the relevant information, and carry out some simple practices. Therefore, reception personnel could play a crucial role in driving migrants through the health pathways and in interacting with health professionals. This type of accompaniment was provided only for some reception centers in the LHA of the study. For this reason, the “G-START” Project has contributed to develope specific diagnostic-therapeutic care pathways dedicated to the ASRs, aimed at getting around the bureaucracy and allowing an easier access to the services.

This study had some limitations. All the migrants’ social groups that were represented in the LHA reception system have been involved. Most of the participants came from Nigeria, so the sample may be slightly biased against the country of origin, but it did reflect the real distribution of the population of interest. Although the saturation of the contents was not reached, the results produced the necessary insight for action. Moreover, some of the individual health needs concerning the personal and intimate spheres, such as victims of trafficking’s health problems, could have emerged in more depth using a different type of data collection, such as in-depth interviews. Furthermore, in the non-neutral environment in which the FGs took place (reception centers) the participants may not have felt totally free to express themselves. The participants’ ability to speak the bridge languages used by the researchers/cultural mediator was in some cases poor. Nevertheless, the FGs strategy made it possible to stimulate discussion among the participants and collect a large amount of data to guide action.

This study raised the attention on health in its bio-psycho-social framework from the ASR’s point of view. At the moment of the project implementation, the available qualitative literature in the Italian setting on this topic was scarce. The multidisciplinary project team played a significant role in the research processes, and also allowed to identify, assess and address all the health issues emerged from the FGs discussion. Furthermore, this qualitative study represents an example of participatory research and ASRs’ involvement in health care planning and services development. It was strongly action-oriented, and its results have contributed to the definition of tailored care pathways, training of health professionals and personnel involved in the reception system, and provision of cultural mediation. All these activities allowed accompanying ASRs to and through the health system, promoting appropriateness of the use of services and the exercise of the right to access. Multilingual peer-to-peer education and communication interventions aimed to capacity building and Health Literacy were also provided (25).

In conclusion, from the results of this study emerged that the ASR population, usually younger and healthier than the local population, presents specific vulnerabilities and health needs that are related to the status of “migrant person.” Having a universal health, education and social system *per se* is not sufficient to guarantee equity, and a set of “personal knowledge and competencies are required in order to access, understand, appraise and use information and services.” This applies not only to ASR population, but also to all citizens that at some point of their existence and for any reason are exposed to higher vulnerability or deprivation.

For these reasons, an effective reorganization of health services driven by a more detailed output analysis of the target population needs is recommended. Developing migrant-sensitive health services and systems requests to consider the own vision and perspective of this population, involve it in health decision-making processes, remove the barriers to the health care access, and enable its personal competencies and Health Literacy. An example of practical actions of reorganization of the health system are represented by the use of cultural mediation, peer to peer education and support, and the training of health professionals at all the levels.

Using qualitative structured tools, like Focus Groups, has been shown to be a valid support to explore health and social needs from the person’s perspective in a participatory way and, subsequently, to guide action. Further research, both qualitative and quantitative, is needed to understand the complex phenomenon of migrants’ bio-psycho-social health, also considering the opportunity to explore the health needs, and the perceived quality of health care from the perspective of Asylum Seekers and, separately, from the point of view of Refugees, considering the differences they may encounter in accessing health services.

At the current time, a Public Health response to migration is an essential dimension in the context of national preparedness. Ensuring the best possible health for migrants in the host countries, like Italy, providing an adequate care that meets their specific needs should represent a priority in order to ensure a more accessible, equitable and effective health care system at local level.

## Data availability statement

The raw data supporting the conclusions of this article will be made available by the authors, without undue reservation.

## Ethics statement

The studies involving human participants were reviewed and approved by “Azienda Sanitaria Locale Roma 5” Ethic Committee. The patients/participants provided their written informed consent to participate in this study.

## Author contributions

AG, AP, and MC contributed to conception and design of the study. AG and FZ conducted the Focus Group. PS organized the database. FM and JP performed data analysis. FM wrote the first draft of the manuscript. FM and AG wrote sections of the manuscript. FM, FZ, AG, AP, JP, PS, GT, AN, MC, EC, LM, SC, FS, and VL read and revising the manuscript critically. All authors contributed to the article and approved the submitted version.

## Funding

The G-START Project has received funding from the European Asylum, Migration and Integration Fund 2014–2020 under grant agreement PROG-2261.

## Conflict of interest

The authors declare that the research was conducted in the absence of any commercial or financial relationships that could be construed as a potential conflict of interest.

## Publisher’s note

All claims expressed in this article are solely those of the authors and do not necessarily represent those of their affiliated organizations, or those of the publisher, the editors and the reviewers. Any product that may be evaluated in this article, or claim that may be made by its manufacturer, is not guaranteed or endorsed by the publisher.

## Abbreviations

LHA: local health authority
ASRs: asylum seekers and refugees
FG: focus group
